# Key subphenotypes of bipolar disorder are differentially associated with polygenic liabilities for bipolar disorder, schizophrenia, and major depressive disorder

**DOI:** 10.1038/s41380-024-02448-1

**Published:** 2024-02-14

**Authors:** Jie Song, Lina Jonsson, Yi Lu, Sarah E. Bergen, Robert Karlsson, Erik Smedler, Katherine Gordon-Smith, Ian Jones, Lisa Jones, Nick Craddock, Patrick F. Sullivan, Paul Lichtenstein, Arianna Di Florio, Mikael Landén

**Affiliations:** 1 Mental Health Center and West China Biomedical Big Data Center, West China Hospital, Sichuan University, Chengdu, China; 2 Department of Medical Epidemiology and Biostatistics, Karolinska Institutet, Stockholm, Sweden; 3 Med-X Center for Informatics, Sichuan University, Chengdu, China; 4 Department of Psychiatry and Neurochemistry, Institute of Neuroscience and Physiology, Sahlgrenska Academy at University of Gothenburg, Gothenburg, Sweden; 5 The Wallenberg Centre for Molecular and Translational Medicine, Gothenburg, Sweden; 6 Department of Clinical Chemistry, Sahlgrenska University Hospital, Gothenburg, Sweden; 7 Psychological Medicine, University of Worcester, Worcester, UK; 8 National Centre for Mental Health, MRC Centre for Neuropsychiatric Genetics and Genomics, Division of Psychological Medicine and Clinical Neurosciences, Cardiff University, Cardiff, UK; 9 Three Counties Medical School, University of Worcester, Worcester, UK; 10 Department of Genetics and Psychiatry, University of North Carolina, Chapel Hill, NC USA

**Keywords:** Genetics, Bipolar disorder

## Abstract

Bipolar disorder (BD) features heterogenous clinical presentation and course of illness. It remains unclear how subphenotypes associate with genetic loadings of BD and related psychiatric disorders. We investigated associations between the subphenotypes and polygenic risk scores (PRS) for BD, schizophrenia, and major depressive disorder (MDD) in two BD cohorts from Sweden (*N* = 5180) and the UK (*N* = 2577). Participants were assessed through interviews and medical records for inter-episode remission, psychotic features during mood episodes, global assessment of functioning (GAF, function and symptom burden dimensions), and comorbid anxiety disorders. Meta-analyses based on both cohorts showed that inter-episode remission and GAF-function were positively correlated with *BD-PRS* but negatively correlated with schizophrenia-PRS (*SCZ-PRS)* and *MDD-PRS*. Moreover, *BD-PRS* was negatively, and *MDD-PRS* positively, associated with the risk of comorbid anxiety disorders. Finally, *SCZ-PRS* was positively associated with psychotic symptoms during mood episodes. Assuming a higher PRS of certain psychiatric disorders in cases with a positive family history, we further tested the associations between subphenotypes in index BD people and occurrence of BD, schizophrenia, or MDD in their relatives using Swedish national registries. BD patients with a relative diagnosed with BD had: (1) higher GAF and lower risk of comorbid anxiety than those with a relative diagnosed with schizophrenia or MDD, (2) lower risk of psychotic symptoms than those with a relative diagnosed with schizophrenia. Our findings shed light on the genetic underpinnings of the heterogeneity in clinical manifestations and course of illness in BD, which ultimately provide insights for developing personalized approaches to the diagnosis and treatment.

## Introduction

Ever since Kraepelin distinguished manic-depressive illness from dementia praecox, the hallmark of bipolar disorder (BD) has been disruptive episodes of mania and depression between which patients recover and regain function [1]. Persons with BD may experience psychotic symptoms during mood episodes but not during euthymic periods [2]. This course of illness contrasts the prototypical form of schizophrenia—which Kraepelin called dementia praecox—that follows a chronic, deteriorating course. Although psychotic symptoms may wax and wane in schizophrenia, negative symptoms persist and full recovery is rare. More recently, the distinguishing feature of hypomanic or manic episodes was used to separate recurrent unipolar depression from BD [3]. Comorbid anxiety disorder is more common in unipolar depression that in BD [4].

Although these characteristics distinguishing between BD, schizophrenia, and recurrent unipolar depression remain in modern diagnostic classification systems, the actual clinical presentation and natural course of BD varies considerably among individuals [5]. While many patients with BD do indeed regain full function between mood episodes (*complete inter-episode remission*), a significant portion suffer from residual mood symptoms or lingering cognitive impairment that prevent full functional recovery [6, 7]. Likewise, some but not all BD patients feature psychotic symptoms during mood episodes, or suffer from comorbid anxiety disorders.

Genome-wide association studies (GWAS) and post-GWAS analyses have found common risk variants shared between BD, schizophrenia, and major depressive disorder (MDD) that help explain the overlap in symptom presentations [8–11]. It has been less studied to what extent genetic factors differentially influence features within a diagnostic category. In recent years, polygenic risk scores (PRS) have been widely applied to study the association between complex genetic traits and clinical symptoms. Studies of BD have reported that the polygenic loading of schizophrenia, as measured by PRS, is negatively associated with lithium response and age of onset, but positively associated with psychotic features [12–15]. Moreover, BD patients with a higher genetic liability of MDD respond worse to lithium [16]. Finally, a recent study found that the *MDD-PRS* was strongly associated with the depression dimension, whereas the schizophrenia PRS (*SCZ-PRS*) was strongly associated with the psychosis dimension among BD patients [17].

To reconcile the categorical distinctions at the core of the current psychiatric diagnostic systems—such as the Kreapelinian dichotomy and the differentiation between BD and unipolar depression—with the evolving comprehension of these disorders being dimensional and having polygenic underpinnings, we investigated how polygenic liabilities of BD, schizophrenia, and MDD are associated with the following key subphenotypes of BD: inter-episode remission, global functioning and symptom burden, psychotic features during mood episodes, and comorbid anxiety disorders. We performed a BD case-only study and tested the associations between the three psychiatric disorders’ PRS and these BD subphenotypes in a Swedish cohort and a UK cohort, with subsequent meta-analyses. Assuming a higher PRS of certain psychiatric disorders in cases with a positive family history, we also examined the features of subphenotypes in a familial coaggregation design using Swedish national registries, where BD index persons who had a relative with BD were compared with BD index persons who had a relative with schizophrenia or MDD.

## Methods

### Subjects

BD cases were enrolled in the Swedish Bipolar Collection study (SWEBIC), to which most participants had been recruited via the Swedish National Quality Register for BD (BipoläR) [18]. Patients were diagnosed according to the DSM-IV-TR with BD type 1, BD type 2, BD not otherwise specified (BD-NOS), or schizoaffective disorder bipolar type. BipoläR captures basic demographic data for each individual along with interventions and outcomes. Outpatient clinics can register patients in BipoläR at any time point during the course of illness. A small number of cases and controls (*N* = 284) were enrolled through the St. Göran Bipolar Project—whose work-up procedures have been previously described elsewhere [19–21]—but were all also included in BipoläR. SWEBIC study participants were recruited up until December 2013 and more than 5000 patients volunteered to participate. All ascertainment procedures were approved by the Regional Ethical Review Board in Stockholm, Sweden, and all participants provided written informed consent.

For the familial coaggregation study, we established a study population of biological relatives to index BD persons by linking the quality register BipoläR to Swedish National Registries [22]. The first-degree (parent, offspring, and full sibling) and second-degree relatives (grandparents, grandchildren, aunt/uncle, nephew/niece, maternal and paternal half-sibling) were identified through the Multi-Generation Registry. The lifetime diagnoses of BD, schizophrenia, and MDD in relatives to BD index persons were identified from the National Patient Registry where records of psychiatric inpatient discharges are available since 1973 and outpatient visits in specialized psychiatric care since 2001. Diagnoses for BD, schizophrenia, and MDD were coded according to the International Classification of Diseases (ICD) with a hierarchical approach (Supplementary Table S1). Individuals’ sex and year of birth were obtained from the Total Population Registry. All registries were followed from their start until December 2013. The study was approved by the regional ethics committee in Stockholm.

Cases from the UK were obtained from the Bipolar Disorder Research Network (BDRN) study, an ongoing program of research into the genetic and environmental causes of BD and related mood disorders. Detailed description of the program can be found in prior publications [23, 24]. Briefly, participants were recruited via community mental health teams, advertisements in the media, and through patient support organizations across the UK. Participants were ≥ 18 years old, met DSM-IV diagnostic criteria for main lifetime diagnosis of BD subtype 1 and 2, schizoaffective disorder of bipolar type, and BD-NOS, and provided written informed consent. A total of 2577 BD cases remained after genotyping quality control in the genotyping wave we received in 2014. The BDRN study has approval from the West Midlands NHS Research Ethics Committee (MREC/97/7/01) and all participating NHS Trusts and Health Boards.

### Subphenotype definitions

The BD subphenotypes studied were inter-episode remission, global assessment of functioning (GAF; function and symptom burden dimensions for Swedish cohort, and function dimension for the UK cohort), psychotic symptoms during mood episodes, and comorbid anxiety disorders. [25] In the Swedish cohort, subphenotypes were obtained from three sources: BipoläR (with patients’ information registered at outpatient clinics), telephone interviews conducted by trained research nurses, and the National Patients Registry. In the UK cohort, subphenotypes were derived from clinical assessments done retrospectively by SCAN interview (assessed when the participants’ moods were stable) and available case-notes review. [26] A summary of definitions and sample sizes for each subphenotype are shown in Tables 1–2.

**Table 1:** Definition of subphenotypes in BD.

	SWEBIC (*N* = 5180)	BDRN (*N* = 2577)
Inter-episode remission	Collected at the SWEBIC telephone interview. The question was first introduced as follows: “Bipolar disorder is said to be an episodic illness. One can have depressive, hypomanic, manic, or mixed episodes. Have you had depressions or episodes with elated mood?” Provided that the respondent answered yes, the follow-up question read: “How have you been between these episodes?” (if the patient reported more than one episode) or “How have you been since your last episode?” (if the patient had only had one mood episode). The response alternatives were: (i) “Completely recovered, back to normal, became well, working or studying, I am doing well now”, (ii) “Never fully recovered to the functioning prior to illness debut, have remaining difficulties such as need to work part time, (iii) “Not recovered, chronically functionally impaired, not able to work, being on long term sickness leave or receiving disability pension”, (iv) “Don’t know”, (v) “Don’t want to answer”. This variable is unavailable in familial coaggregation analysis.	The inter-episode remission was evaluated by interview and case-note review with OPCRIT item number 90 (i.e., course of disorder) categorized as: (i) Good remission, (ii) Partial remission, (iii) No remission, and (iv) Unknown. [52] To generate more balanced subgroups, we merged partial and no remission because only 12 individuals reported ‘no remission’.
Global assessment of functioning (GAF)	GAF (1–100) was collected from BipoläR where treating physicians rates the GAF-symptom and GAF-function dimensions. [25] We used the mean GAF during the follow-up.	GAF-function was rated by selecting the lowest range that best described the functioning during the last week before interview as assessed by the interviewer.
Psychotic symptom during mood episodes	History of psychotic symptoms during mood episodes was collected from the SWEBIC telephone interview. The question read: “Have you ever lost touch with reality and had psychotic symptoms, i.e., heard or seen things that other people did not see or hear, experienced things that you later on realized were not real?” The raters were asked to weigh in their clinical judgement and instructed to code an uncertain response, or response that the rater did not consider psychotic (i.e., depersonalization/ derealization experiences) as ‘no’. In familial coaggregation analysis, this phenotype was identified in the National Patient Registry, with ICD-10 codes displayed in supplementary Table S1.	The lifetime history of any psychosis during mood episodes was recoded as Yes or No using data from the interview and case-note review.
Comorbid anxiety disorders	Anxiety disorders were identified in the National Patient Registry, with ICD codes displayed in supplementary Table S1.	The lifetime presence of known anxiety disorder was defined as the presence of a doctor diagnosis of any anxiety disorder recorded in the medical case-notes or reported at interview, or significantly impairing anxiety episodes ascertained during the SCAN interview.

Interviews of subphenotypes in SWEBIC cohort were all performed in Swedish and the text was translated in English in the table.

**Table 2:** Descriptive statistics of patients with BD.

Characteristics		SWEBIC (*N* = 5180)		BDRN (*N* = 2577)		*P* value of statistic tests between groups
		*N* available	Statistics	*N* available	Statistics	
Sex (Female %)		5180	62.2	2577	69.4	4.67 × 10^−10^
Birth year (Mean ± SD)		5064	1959 ± 15	2564	1963 ± 12	1.99 × 10^−^^26^
BD subtype		4931		2535		
* BD1 (%)*		2287	46.4	1646	64.9	3.98 × 10^−^^189^
* BD2 (%)*		1704	34.6	795	31.4	
* Schizoaffective disorder of bipolar type (%)*		65	1.3	94	3.7	
* BD NOS (%)*		875	17.7	0	0	
Inter-episode remission		4231		2369		
	*Full remission (%)*	1878	44.4	1289	54.4	2.65 × 10^−168^
	*Partial remission (%)*	1199	28.3	1068	45.1	
	*No remission (%)*	1154	27.3	12	0.5	
GAF-Function (Mean ± SD)		4013	68.0 ± 11.9	2517	78.2 ± 9.6	4.89 × 10^−263^
GAF-Symptom (Mean ± SD)		4015	67.2 ± 11.2	NA	NA	NA
Psychotic symptom during mood episodes (%)		4200	47.9	2132	58.4	2.89 × 10^−15^
Comorbid anxiety disorders (%)		5077	40.2	2328	77.8	1.57 × 10^−198^

The sample sizes of individuals with available information on the subphenotypes were listed as “N available”. Statistics are percentages for categorical variables (N of subphenotypes divided by N available) and mean ± SD for continuous variables. Statistical comparisons are *t* test for continuous variables and chi-square test for categorical variables. All statistical comparisons exceed Bonferroni correction (*N* = 7, *P* < 0.007).

*SD* standard deviation, *BD1* bipolar disorder subtype 1, *BD2* bipolar disorder subtype 2, *BD NOS* bipolar disorder not otherwise specified, *GAF*-*function* global assessment of functioning, function dimension, *GAF-symptom* global assessment of functioning, symptom dimension, *NA* not applicable.

### Genotyping and quality control (QC)

DNA collection and genotyping procedures in SWEBIC has been previously described [27]. In brief, DNA was extracted from whole blood samples stored at the Karolinska Institutet Biobank. Genotyping was conducted at the Broad Institute of Harvard and MIT using Affymetrix 6.0 (wave 1, Affymetrix, Santa Clara, CA, USA), Illumina OmniExpress (wave 2, Illumina, San Diego, CA, USA), and Illumina PsychArray-24 v1.2 (wave 3, Illumina, San Diego, CA, USA). The data genotyped on different arrays were processed in the Psychiatric Genomics Consortium (PGC) RICOPILI pipeline for QC and imputation [28]. Ancestry outliers were identified using data from 1000 Genome Project (Phase 3 version 5) [29]. The final sample contained 5458 cases of which 5180 had at least one recorded phenotype of interest. The QC exclusionary parameters for subjects were: genotype missingness rate >5%, ancestry outliers identified via multidimensional scaling (MDS), suspected sample error or contamination (i.e., subject heterozygosity rate >10%), ambiguous genetic sex, and a randomly selected member of any pair of subjects identified as related (pairwise pi-hat >0.20). Exclusionary parameters for single nucleotide polymorphisms (SNPs) were: marked deviations from Hardy-Weinberg equilibrium (*P* < 1 × 10^−6^), SNP missingness rate >5%, minor allele frequency (MAF) < 1%, differential missingness based on affection status (*P* < 1 × 10^−6^), and differential missingness based on haplotype (*P* < 1 × 10^−10^). Following the QC steps, imputation was performed by first pre-phasing the data using SHAPEIT2 and then imputing using IMPUTE2 with the 1000 Genomes Project integrated variant set (Phase 1, released March 2012) as the reference panel [30–32].

In the UK sample, DNA was extracted from whole blood at the neuropsychiatric genetics laboratory at Cardiff University. DNA were genotyped using Illumina OmniExpress and Illumina ComboChip. Genotyping QC and imputation for UK samples is described in detail in previous publications. [27, 33] Briefly, the QC exclusionary parameters for the BDRN sample were: subject missingness rate >2%, ambiguous genetic sex, subject heterozygosity rate >15%, SNP missingness rate >2%, MAF < 1%, marked departure from Hardy-Weinberg equilibrium (*P* < 5 × 10^−5^), differential missingness based on haplotype (*P* < 1 × 10^−10^) between cases and controls and differential missingness for SNPs (*P* < 1 × 10^−3^), population outliers identified via MDS, and a random member of each pair of related subjects (pairwise pi-hat >0.10). Datasets after QC were then pre-phased using SHAPEIT and imputed with the 1000 Genomes Project integrated variant set (Phase 1, released March 2012) as the reference panel using IMPUTE2 [30–32].

### Polygenic risk score profiling

The most recent and largest GWAS for schizophrenia and BD performed by PGC were used as discovery sets for SNP selection and risk allele weighting for generating PRS [13, 34, 35]. For *MDD-PRS* discovery set, we used the GWAS summary statistics of MDD in 2018 excluding subjects from *23andMe* [36]. We did not use the most recent MDD GWAS [37] because the publicly available summary statistics lack the imputation INFO score, and we wanted to avoid possible sample overlap between the UK biobank and the BDRN sample. We used the summary statistics of meta-analyzed GWAS with European ancestry only and performed GWAS meta-analyses after removing SWEBIC or BDRN samples to resolve sample overlap between training and testing sets. To further evaluate the specificity and potential overlap across the genetic liabilities of mental-related traits, we also generated PRS for anxiety disorders, neuroticism, and educational attainment using the most recent GWAS data available [38–40]. This was done in consideration of their potential relationship to the BD subphenotypes under investigation.

For PRS calculation, we applied PRS-CS (version Aug 10, 2023) that generates posterior variant effects accounting for linkage disequilibrium (LD) structure and genetic architecture [41], which outperforms the standard pruning and thresholding (P + T) method. PRS-CS was run with default values for parameters and automatic estimation of the global shrinkage parameter values *phi* per chromosome, with a specified seed for random number generation for reproducibility. The total sample size of each discovery GWAS was used for the sample size parameter. We used the “best guess” genotype hardcall files filtered on imputation quality (INFO score >0.8) as the bim file for target dataset to provide a list of available variants. The GWAS meta-analyses above were used to train PRS-CS through the LD reference panel constructed using the 1000 Genomes Project phase 3, European reference [29]. The genetic markers were further restricted to those present in the HapMap3 reference sample [42]. PRS were then generated for each individual as the sum of the imputed SNP dosages weighted by the PRS-CS posterior allele effect using PLINK1.9 [43]. All PRS were standardized using z-score transformations to have mean zero and unit variance within each target set (i.e., within each wave in SWEBIC cohort and within BDRN cohort) to account for variation in SNP numbers used for PRS calculation.

### Association tests between PRS of major psychiatric disorders and BD subphenotypes

We tested if PRS of BD, schizophrenia and MDD were associated with BD subphenotypes using regression models fit for the respective outcome. In SWEBIC cohort, we applied ordinal logistic regression for the inter-episode remission variable, logistic regressions for the psychotic symptoms and comorbid anxiety disorder variables, and linear regressions for GAF-function and GAF-symptom variables. In the BDRN cohort, the same models (as in SWEBIC) were applied for psychotic symptoms, comorbid anxiety disorders, and GAF-function. For inter-episode remission, we merged ‘partial remission’ and ‘no remission’—because only 12 individuals reported ‘no remission’—and used a logistic regression model. Moreover, we did not test for GAF-symptom since this phenotype was unavailable in the UK sample. PRS were used as continuous variables in all analyses with adjustment for sex, birth year, the first six ancestry principal components, and genotyping platforms. All three PRS were tested jointly in the model. Additionally, we performed association tests stratified by the BD subtypes 1 and 2. Psychotic symptoms during mood episodes was not tested for BD2 cohort because BD2 cases did not have this subphenotype. Finally, we performed random-effect meta-analyses combining the results from the two cohorts for all patients with BD, BD1 and BD2, respectively. As a sensitivity test to evaluate the specificity of PRS effects for the three major psychiatric disorders on BD subphenotypes, we conducted additional analyses by including the PRS of anxiety disorders, neuroticism, and educational attainment in the models for all BD cases. We used the random-effect model to obtain more conservative estimates as it accounts for uncertainties resulting from heterogeneity across the two cohorts. The Bonferroni method was used to correct for multiple testing, with respective number of tests corrected for (indicated under each result table). All association tests were performed using R (v4.0.3) [44] and the meta-analyses were performed using package *metafor* [45].

### Familial coaggregation between BD subphenotypes and major psychiatric disorders in relatives

Assuming that individuals with a family history of BD, schizophrenia, or MDD would have a higher genetic loading of the corresponding psychiatric disorder, we performed familial coaggregation analyses to examine the magnitude of subphenotype occurrence (or levels of continuous subphenotype measures, i.e., GAF) in BD index person with relatives of BD, schizophrenia and MDD. We investigated GAF-function, GAF-symptom, as well as diagnoses of psychotic and anxiety disorders in index persons with BD (see Supplementary Table S1 for ICD codes). We restricted our analysis to cohorts of relatives diagnosed with either BD, schizophrenia, or MDD, and to index BD cases who had data on the respective subphenotype. We used a linear regression model to estimate the difference in GAF-function/symptom between index BD cases with schizophrenia relatives and index BD cases with BD relatives. Logistic regression models were used to estimate the odds ratios (ORs) of a comorbid anxiety disorder and psychotic symptoms in index BD cases who had relatives with schizophrenia compared with those whose relatives had BD. The same analyses were repeated in BD individuals who had relatives with MDD compared with those whose relatives had BD. To increase power, we estimated the coefficients by combining all first- and second-degree relatives. We adjusted for sex, year of birth category (before 1955, 1955–1962, 1963–1969, 1970–1977, 1978–1988, after 1988), and biological relatedness (first- and second-degree relatives) to the index individuals. We calculated the 95% confidence intervals (CIs) with robust standard errors to account for the non-independence between individuals due to familial clustering. The subphenotype ‘inter-episode remission’ was not tested because SWEBIC telephone interview data could not be linked to the Multi-Generation Registry due to ethical restraints. SAS 9.4 (SAS Institute, Cary, NC) was used for data management and analysis for this part [46].

## Results

The descriptive characteristics for SWEBIC and BDRN study participants are shown in Table 2. The two cohorts differ in several aspects, including sex ratio, frequencies of BD subtypes and the prevalence of the four studied subphenotypes, which is expected considering different sample recruitments and different subphenotype assessments. Of note, we observed a higher percentage of BD1 in BDRN, which is likely because cases used in this study were mainly recruited via mental health services for the International Cohort Collection of Bipolar Disorder consortium [33]. Moreover, the few BD-NOS cases were removed due to the low frequency after genotyping QC in BDRN. The separate characteristics in BD1 and BD2 cases was available in Supplementary Table S2.

**Fig. 1:**
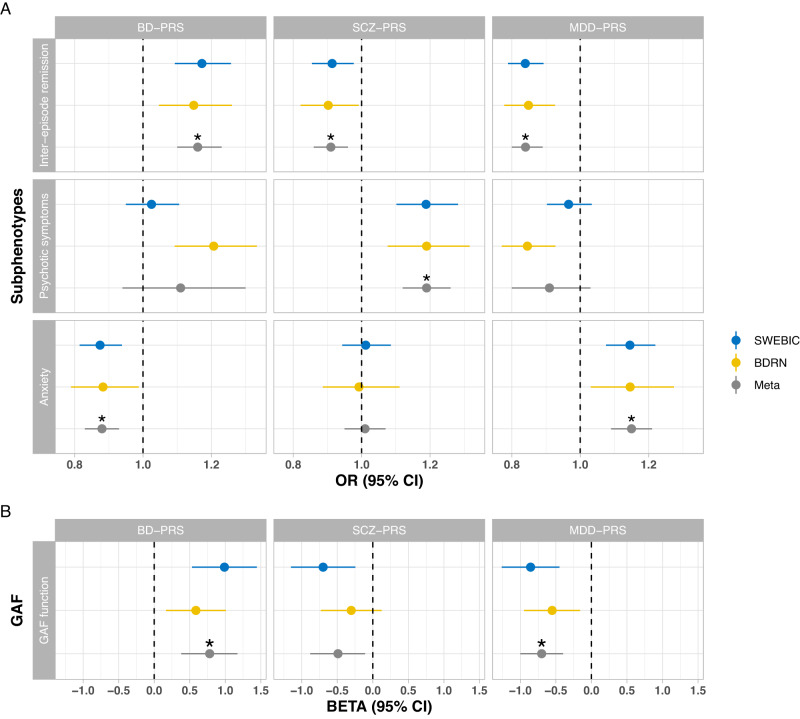
Association between subphenotypes in BD patients and polygenic risk scores of BD, schizophrenia and MDD in Sweden SWEBIC, UK BDRN cohort and meta-analyses. Genetic profiles include polygenetic risk scores for bipolar disorder (*BD-PRS*), schizophrenia (*SCZ-PRS*), and major depressive disorder (*MDD-PRS*). **A** Logistic regression was applied in analyses for psychotic symptoms and comorbid anxiety in both cohorts. For inter-episode remission, ordinal logistic regression was applied in SWEBIC cohort and logistic regression was applied in BDRN cohort. Odds ratios (OR) and 95% confidence intervals (CI) are reported. **B** Linear regression was applied for GAF function and GAF symptom (for SWEBIC only), and Beta and 95% CI are reported. GAF symptom was not available for BDRN and meta-analysis and was not present in this figure. In each cohort, analysis models included all three PRS and were adjusted for sex, birth year, the first six population principal components, and genotyping platforms. Random-effect meta-analyses were performed and *P* < 0.004 (corrected for 12 tests in the meta-analyses) are considered significant and marked with asterisk. The data for this figure are in Supplementary Table S3.

The associations between PRS for the three psychiatric disorders and each BD subphenotype are shown in Fig. 1 and Supplementary Table S3. For *BD-PRS*, we observed similar trends of associations with BD subphenotypes in SWEBIC and BDRN cohorts, except for psychotic symptoms during mood episodes that showed a positive correlation with *BD-PRS* in BDRN (OR 1.21, 95%CI 1.09–1.33, *P* = 2.12 × 10^−4^) but no correlation in the SWEBIC cohort (OR = 1.02, *P* = 0.53). Meta-analyses based on both cohorts yielded no significant heterogeneities for other subphenotypes. *BD-PRS* was positively associated with complete inter-episode remission (OR 1.16, 95%CI 1.10–1.23, *P* = 1.05 × 10^−7^). This means that the odds of complete vs. incomplete inter-episode remission increases by 16% (95%CI 10–23%) with each increasing unit of *BD-PRS* given the same *SCZ-PRS* and *MDD-PRS*. Further, *BD-PRS* associated positively with GAF-function (beta 0.78, 95%CI 0.38–1.17, *P* = 1.06 × 10^−4^), meaning that higher *BD-PRS* predicted higher global functioning. Finally, higher *BD-PRS* was associated with lower risk of comorbid anxiety disorders (OR 0.88, 95%CI 0.83–0.93, *P* = 1.60 × 10^−5^). Additionally, in the SWEBIC cohort, higher *BD-PRS* was also associated with lower global symptoms (beta for GAF-symptom 0.96, 95%CI 0.53–1.38, *P* = 1.14 × 10^−5^).

For *SCZ-PRS*, in both cohorts, we found a negative association with full inter-episode remission and a positive association with higher likelihood of psychotic symptoms during mood episodes, with meta-analyzed OR estimates of 0.91 (95%CI 0.86–0.96, *P* = 6.98 × 10^−4^) and 1.19 (95%CI 1.12–1.26, *P* = 2.07 × 10^−8^), respectively. The association for the GAF-function did not survive correction for multiple testing (beta −0.49, 95%CI −0.88 – −0.11, *P* = 0.01, Fig. 1 and Table S3). Notably, however, the estimates of correlations between *SCZ-PRS* and GAF were all negative in both cohorts (GAF-function and GAF-symptom in SWEBIC, GAF-function in BDRN, see Fig. 1 and Table S3).

Regarding *MDD-PRS*, we found negative associations with full inter-episode remission (meta OR 0.84, 95%CI 0.80–0.89, *P* = 2.78 × 10^−11^) and GAF-function (meta beta −0.70, 95%CI −1.00 – −0.40, *P* = 3.76 × 10^−6^) and a positive association with the risk of comorbid anxiety disorders (meta OR 1.15, 95%CI 1.09–1.21, *P* = 8.73 × 10^−^^7^), with no significant heterogeneity between the two study cohorts. Further, *MDD-PRS* was negatively associated with GAF-symptom in the SWEBIC cohort. While *MDD-PRS* was found to be negatively associated with psychotic symptoms in the BDRN cohort, no association was found in the SWEBIC cohort or in the meta-analysis, with a Heterogeneity test *P* = 0.02.

In the sensitivity analysis where we tested the PRS effects of six traits jointly (BD, schizophrenia, MDD, anxiety disorders, neuroticism, and educational attainment), we observed similar results for the PRS of the three major psychiatric disorders, except that the associations for *MDD-PRS* with comorbid anxiety and GAF-function were no longer significant after multiple testing correction (*P* = 0.02 and 0.002, respectively; see Supplementary Table S4). Additionally, we found that neuroticism-*PRS* was associated with a higher risk of comorbid anxiety, while PRS for educational attainment was positively associated with complete inter-episode remission and with psychotic symptoms during mood episodes.

**Fig. 2:**
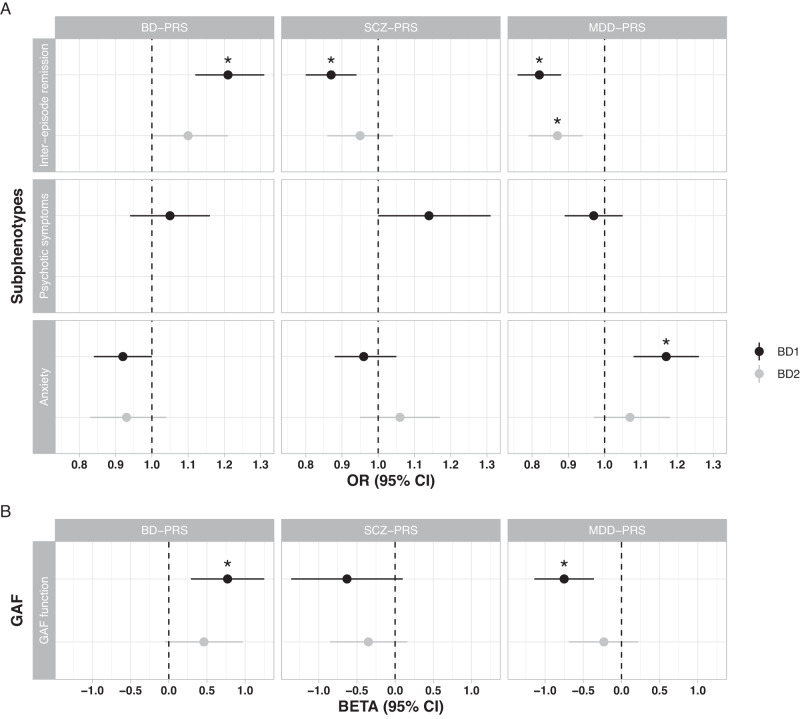
Association between subphenotypes in BD patients and polygenic risk scores of BD, schizophrenia and MDD in BD1 and BD2 cases in meta-analyses. Genetic profiles include polygenetic risk scores for bipolar disorder (*BD-PRS*), schizophrenia (*SCZ-PRS)*, and major depressive disorder (*MDD-PRS*). The results of association tests for each cohort and meta-analyses are in Supplementary Tables S5–6, among which the results of meta-analyses were presented here. The regression models were the same as in the main analyses, with odds ratios (OR) reported for categorical variables in Panel A and Beta reported for continuous variable in Panel B. Psychotic symptoms during mood episodes was not tested because BD2 cases did not have this subphenotype. *P* < 0.004 (corrected for 12 tests in BD1 meta-analyses) and *P* < 0.005 (corrected for 9 tests in BD2 meta-analyses) are considered significant and marked with asterisk.

The results for association tests stratified by the BD subtypes 1 and 2 are shown in Fig. 2 (meta-analyses results) and Supplementary Tables S5–6. Among BD1 cases, the results remained similar compared to the main analyses, although the associations between *BD-PRS* and anxiety as well as *SCZ-PRS* and psychotic symptoms were not statistically significant (*P* = 0.04 and 0.05 in the meta-analyses, respectively). Among BD2 cases, the magnitude of the associations attenuated, and only the association between *MDD-PRS* and inter-episode remission remained statistically significant (meta OR 0.87, 95%CI 0.79–0.94, *P* = 0.001).

**Fig. 3:**
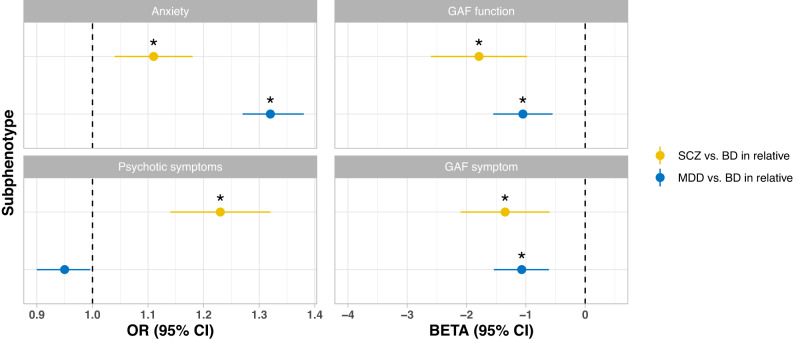
Association between subphenotypes in index person with BD and lifetime diagnosis of three major psychiatric disorders in the first- and second-degree relatives. Logistic regression was applied in analyses for psychotic symptoms and comorbid anxiety. Odds ratios (OR) and 95% confidence intervals (CI) are reported. Linear regression was applied for GAF function and GAF symptom, and Beta and 95% CI are reported. Models were adjusted for sex, categorical year of birth and degree of biological relatedness. For anxiety and psychotic symptom (which was extracted from the Swedish national registries), models were additionally adjusted for anxiety or psychotic symptoms in the relatives. Estimates past significance threshold (corrected for 8 tests, *P* < 0.006) are marked with asterisk. The data for this figure are shown in Supplementary Table S7.

The familial coaggregation analyses between psychiatric disorders in relatives and subphenotypes (GAF, psychotic symptoms, and anxiety disorders) in index BD cases are shown in Fig. 3 and Supplementary Table S7. First, index BD cases with a relative diagnosed with schizophrenia or MDD had lower GAF-function and GAF-symptom than index BD cases with a relative diagnosed with BD. Second, anxiety disorders were more prevalent in index BD cases who had a relative diagnosed with schizophrenia or MDD compared with BD index cases who had a relative diagnosed with BD. Third, compared with BD cases who had a relative diagnosed with BD, the risk of having been diagnosed with a psychotic disorder was higher in BD cases who had a relative diagnosed with schizophrenia.

## Discussion

Combining two large BD cohorts in Sweden and the UK, we found distinct associations between key BD subphenotypes and genetic loading for BD, schizophrenia, and MDD. Most notably, full inter-episode remission and GAF were positively correlated with *BD-PRS*, but negatively correlated with *SCZ-PRS and MDD-PRS*. Moreover, *BD-PRS* was negatively, and *MDD-PRS* positively, associated with the risk of comorbid anxiety disorders. Finally, *SCZ-PRS* was positively associated with psychotic symptoms during mood episodes. These findings were similar when restricted to the BD1 subtype and were further supported by familial coaggregation analyses using Swedish national registries. Here, GAF scores were higher in BD cases with BD relatives than in BD cases who had schizophrenia or MDD relatives. Further, psychotic and anxiety disorders were less common in BD cases with BD relatives than in BD cases with schizophrenia relatives. Finally, anxiety disorders were less common in BD cases with BD relatives than in BD cases with MDD relatives. Taken together, these findings help explain how genetic factors contribute to the heterogeneous presentations of BD, enhancing our understanding of how polygenic vulnerabilities underpin its multifaceted manifestations.

Intriguingly, these results resonate with the century-old distinction between recurrent manic-depressive disorder (BD) and progressive dementia praecox (later schizophrenia): The finding that complete inter-episode remission was associated with higher *BD-PRS* and lower *SCZ-PRS* echoes the prototypical episodic course of illness of BD and the chronic course of schizophrenia. Likewise, the finding that higher global functioning (as measured with GAF) was associated with higher *BD-PRS* and lower *SCZ-PRS* (borderline significant, *P* = 0.01) aligns with the Kraepelinian notion that schizophrenia features a chronic deteriorating course with persistent impairment, whereas individuals with BD are typically symptom-free when recovered from mood episodes. The divergent influences of genetic liabilities on global psychosocial functioning were further supported by the familial co-occurrence analysis where BD cases with a family history of schizophrenia had lower GAF compared with those with a family history of BD. Taken together, modern polygenetic and epidemiological analyses reflect the historical landmark distinction between recurrent manic-depressive insanity and progressive dementia praecox as outlined by Emil Kraepelin.

The fact that a higher genetic loading of BD was associated with higher psychosocial functioning (i.e., higher GAF-function score)—and lower symptom burden measured by GAF-symptom within the Swedish group—might seem to be counterintuitive. A higher genetic loading of a disease would be assumed to predict a more severe disorder. Such findings might be explained in several perspectives. First, in our cohorts, BD caseness is defined based on the occurrence of mood episodes, without a requirement for long-term impaired functioning or the presence of anxiety outside mood episodes. Indeed, *BD-PRS*, by definition increases the risk for elated mood episodes, has been reported to confer benefits in other domains, for example creativity and educational attainment [35, 47]. Second, the assessment of GAF took place in an outpatient setting, where patients are more likely to be in a state of recovery with fewer symptoms. Higher *BD-PRS* is thus associated with better functioning and fewer symptoms when BD patients are not experiencing acute mood episodes. Notably, this does not mean that higher *BD-PRS* would be associated with less severe (or less frequent) mood episodes, which we did not assess in this study. In fact, both in the SWEBIC sample and in a recent study, *BD-PRS* was associated with higher number of hospitalizations. [48, 49]

Higher *MDD-PRS* associated with less likelihood of inter-episode remission, lower psychosocial function (lower GAF-function score) and higher symptom burden (lower GAF-symptom score, tested in the Swedish sample only), as well as a higher rate of comorbid anxiety disorders. By and large, BD patients with a higher genetic loading of MDD present with worse symptoms and outcomes. The reverse association between *MDD-PRS* and inter-episode remission was not driven by BD2 cases. Moreover, the associations with lower GAF and higher likelihood of anxiety were also supported by the familial co-aggregation analyses using Swedish registry data (i.e., those with MDD relatives had lower GAF and higher risk of anxiety than those with BD relatives). Schizophrenia and MDD are the two psychiatric disorders with the highest genetic correlations with BD [50], but in this regard the BD genetic component seems unique and not shared with the two other psychiatric disorders. PRS derived from schizophrenia and MDD GWAS may thus partly index a general liability for chronic psychopathology severity, as suggested in another study that demonstrates a PRS gradient across schizophrenia and BD subtypes [14].

The observed associations remained significant even after accounting for the PRS of anxiety disorders, neuroticism and educational attainment, though the associations for *MDD-PRS* with comorbid anxiety and GAF-function were attenuated and only borderline significant. This was expected given the high genetic overlap between MDD, anxiety disorders, and neuroticism. The absence of a significant association between PRS for anxiety disorders and comorbid anxiety in BD cases might be partially attributed to the limited sample size (*N* = 17,310) of the discovery GWAS, which did not fully capture the genetic loading. The consistent findings underscore the specificity of PRS for BD, schizophrenia, and MDD in relation to BD subphenotypes, even when considering other correlated PRS. This reinforces our conclusion that, although psychiatric disorders share genetic risk factors, their polygenic liabilities may exert distinct influences on the trajectory and outcome within each disorder. Considering the genetic loading of multiple disorders could provide valuable prognostic insights beyond categorical diagnoses, although it should be noted that the predictive power may be limited for clinical purposes.

Despite somewhat differing characteristics between the two cohorts with respect to sex ratio, subtype frequencies and subphenotype distributions, most results were similar across the SWEBIC and BDRN cohorts. However, we also noted some differences. First, psychotic symptoms during mood episodes were positively associated with *BD-PRS* and negatively associated with *MDD-PRS* in the UK sample but not in the Swedish sample, and significantly heterogenous in the meta-analyses. Notably, however, these across cohort differences were not significant when the analysis was restricted to BD1 cases. Second, although in the same direction and demonstrating no signs of heterogeneity in statistical tests, the magnitudes of associations between three PRS and GAF-function differed. Moreover, we observed a negative association between GAF-function and neuroticism-PRS, and a positive association with education-PRS, in the Swedish sample, while no associations were found in the UK sample. Our result of inter-episode remission is also inconsistent with a recent study that uses OPCRIT item 90 to measure the inter-episode remission among BD cases and reports no relationship with *BD-PRS* [48]. This heterogeneity across studies, which is likely in part due to the different phenotype assessments, limits the generalizability of our findings. For example, the mean of GAF was higher in the BDRN cohort than the SWEBIC cohort (Table 2). This might be because GAF was assessed the week before interview when the participants were well in BDRN, but could occur at any time in SWEBIC. Future investigations in larger cohorts with harmonized phenotype assessments, and comparisons with GAF values from routine clinical samples, are warranted.

The strengths of this study include detailed phenotyping in two large BD cohorts from different countries. Moreover, the PRS analyses using genotype data and familial coaggregation analyses using registry data yield converging evidence between the genetic liability of three psychiatric disorders and BD subphenotypes. The limitations to consider first include that data on inter-episode remission and history of psychotic symptoms were collected during a telephone interview with trained research nurses in the Swedish cohort, the validity of which has not been formally tested. SWEBIC diagnoses and GAF assessments were made by mental health professionals in regular clinical care and not in a controlled research setting. Hence, the subphenotypes in our study cohort, with a mixture of telephone interviews, register data from the quality register *BipoläR*, and registry records from the patient register (using ICD codes) might not be readily generalizable, which may affect attempts to replicate findings. Although we tried to harmonize the phenotype assessments across the Swedish and UK studies, heterogeneity remains and further investigation are warranted. Second, it is challenging to interpret the results of PRS for one disorder and subtypes of the same disorder because “the detailed interpretation depends on the proportion of these subtypes in the discovery sample” [51]. Despite that the interpretation of how *SCZ-* and *MDD-PRS* impact BD subphenotypes is less limited by this constraint, caution is still warranted when interpreting the results for subphenotypes that commonly co-occur in two disorders (e.g., anxiety disorders in MDD and BD). Third, the assessments of subphenotypes should ideally rely on longitudinal assessments, which was not possible for individuals recently diagnosed with BD. Additionally, the determination of GAF differed across cohorts: the Swedish GAF-rating reflects the average scoring gathered during annual follow-up within the BD quality register, while in the UK cohort, GAF was assessed as a one-time evaluation. Although the remaining subphenotypes were based on lifetime history up to the point of interview, study persons are interviewed at a random time point (at which they were relatively well) during their course of illness, and attempts to estimate manifestations of a lifelong illness are subject to uncertainties. To improve precision in subphenotypes, future investigations with extended observation periods are encouraged, as this will enable a more accurate characterization of subphenotypes over time. Fourth, the associations between BD subphenotypes in the index person and three major psychiatric disorders in the relatives are due to shared familial factors, which could be genetic and/or environmental factors. Hence, although the results from our familial analyses provide further support for our genetic findings, there are alternative explanations. Moreover, the definition of psychotic symptoms differs in the PRS and familial analyses (see Table 1). Finally, it should be emphasized that our findings do not suggest that BD patients with a higher genetic loading of BD have a less severe disorder. Rather, these cases are more likely to exhibit typical BD symptoms, including periods of remission and good functioning between mood episodes.

In summary, our study indicates that polygenic liabilities for BD, schizophrenia, and MDD are differently associated with subphenotypes of BD. These findings help explain how genetic factors contribute to the heterogeneous presentations of BD, which ultimately provide insights for developing personalized approaches to the diagnosis and treatment.

## Supplementary information

Supplementary information

## Data Availability

Custom written R and SAS scripts used for statistical analyses can be provided upon request. The pipeline to compute polygenic scores can be found on GitHub (https://github.com/getian107/PRScs). Due to data protection regulations, we are not allowed to share individual-level register data with a third party. However, Swedish register data used in this study can be applied from Statistics Sweden (https://www.scb.se/en/), the Swedish National Board of Health and Welfare (Socialstyrelsen, https://www.socialstyrelsen.se/en/), and the Centre of Register Västra Götaland (https://registercentrum.se/in-english/centre-of-registers-vaestra-goetaland/p/H1nJ_MX3z). GWAS summary statistics are publicly available through their respective consortia (Psychiatric Genomics Consortium, https://pgc.unc.edu/, Social Science Genetic Association Consortium (SSGAC), http://www.thessgac.org/data, and https://ctg.cncr.nl/software/summary_statistics).
