# Supplementary material for: Key subphenotypes of bipolar disorder are differentially associated with polygenic liabilities for bipolar disorder, schizophrenia, and major depressive disorder
Source: Mol Psychiatry. 2024 Feb 14;29(7):1941–50. doi: 10.1038/s41380-024-02448-1 (PMC11408248; doi:10.1038/s41380-024-02448-1)
Supplement: Supplementary file 1 — Supplementary information [file 41380_2024_2448_MOESM1_ESM.docx]

**Supplementary Materials**

**Supplementary Table S1.** **Definition of psychiatric disorders in familial coaggregation analyses**

| **Psychiatric disorder** | **ICD 8 (1969-1986)** | **ICD 9 (1987-1996)** | **ICD 10 (1997-)** |
| --- | --- | --- | --- |
| Anxiety disorders | 300 except 300.4 | 300 except 300E | F40-F42, F44-F45, F48 |
| Schizophrenia | 295, 2950, 2951, 2952, 2953, 2954, 2956, 2957, 2958, 2959 | 295, 295A, 295B, 295C, 295D, 295E, 295G, 295H, 295W, 295X | F20, F200, F201, F202, F203, F204, F205, F206, F208, F209, F25, F250, F251, F252, F258, F259 |
| Bipolar disorder | 2961, 2962, 2963 | 296A, 296C, 296D, 296E, 296F, 296G, 296H, 296W, 296X | F30, F301, F302, F308, F309, F31, F310, F311, F312, F313, F314, F315, F316, F317, F318, F319 |
| Major depression disorder | 2960, 300E | 296B, 311 | F32, F320, F321, F322, F323, F328, F329, F33, F330, F331, F332, F333, F334, F338, F339, F34, F348, F349, F38, F380, F381, F388, F39 |
| Bipolar disorder with psychotic symptoms | NA | NA | F20-F29, F30.2, F31.2, F31.5, F32.3 |

Individuals with any diagnosis of bipolar disorder but not schizophrenia were regarded as having bipolar disorder; individuals with an episode of major depression disorder but neither schizophrenia nor bipolar disorder were coded as having major depression disorder. Bipolar disorder with psychotic symptoms are those that apart from bipolar disorder have ever been diagnosed with a psychotic disorder (F20-F29) and those with psychotic mania (F30.2 or F31.2) or psychotic depression (F31.5 or F32.3).

**Supplementary Table S2. Descriptive statistics for patients with BD1 and BD2 subtypes**

| **Characteristics** | | **SWEBIC** | | **BDRN** | |
| --- | --- | --- | --- | --- | --- |
|  |  | **BD1 (N=2,287)** | **BD2 (N=1,704)** | **BD1 (N=1,646)** | **BD2 (N=795)** |
| **Sex (Female %)** | | 1,318 (57.6) | 1,168 (68.5) | 1,155 (70.2) | 555 (69.8) |
| **Birth year (Mean** **± SD)** | | 1958±14 | 1959±15 | 1962±11 | 1964±12 |
| **Inter-episode remission** | |  |  |  |  |
|  | ***Full remission (%)*** | 864 (37.8) | 637 (37.8) | 854 (51.9) | 409 (51.4) |
|  | ***Partial remission (%)*** | 496 (21.7) | 466 (27.3) | 660 (40.1) | 346 (43.5) |
|  | ***No remission (%)*** | 519 (22.7) | 389 (22.8) | 12 (0.4) | 1 (0.4) |
|  | ***NA (%)*** | 408 (17.8) | 212 (12.4) | 125 (7.3) | 39 (4.9) |
| **GAF-Function (Mean ± SD)** | | 68.5±12.3 | 68.1±11.4 | 78.7±9.4 | 77.7±9.0 |
| **GAF-Symptom (Mean ± SD)** | | 68.1±11.4 | 66.8±10.8 | - | - |
| **Psychotic symptom during mood episodes (%)** | |  |  |  |  |
| ***Yes (%)*** | | 1176 (51.4) | - | 1,070 (65.0) | - |
| ***No (%)*** | | 695 (30.4) | - | 345 (21.0) | - |
| ***NA (%)*** | | 416 (18.2) | - | 231 (14.0) | - |
| **Comorbid anxiety disorders (Yes, %)** | |  |  |  |  |
| ***Yes (%)*** | | 788 (34.5) | 748 (43.9) | 1,125 (68.3) | 623 (78.4) |
| ***No (%)*** | | 1,499 (65.5) | 955 (56.0) | 386 (23.5) | 114 (14.3) |
| ***NA (%)*** | | 0 (0) | 1 (0) | 135 (8.2) | 58 (7.3) |

SD: standard deviation; BD1: bipolar disorder subtype 1; BD2: bipolar disorder subtype 2; GAF-function: global assessment of functioning, function dimension; GAF-symptom: global assessment of functioning, symptom dimension. Phenotype definitions are in Table 1 in the main text. Psychotic symptom is not recorded in BD2 cases. Statistics are percentages for categorical variables and mean ± SD for continuous variables (after removing individuals with missing values).

**Supplementary Table S3. Association between subphenotypes in patients with bipolar disorder and polygenic risk scores of schizophrenia (*SCZ-PRS*), bipolar disorder (*BD-PRS*), and major depressive disorder (*MDD-PRS*)**

| **PRS** | **BD subphenotypes** | **coefficient** | **SWEBIC** | | **BDRN** | | **Meta** | | | |
| --- | --- | --- | --- | --- | --- | --- | --- | --- | --- | --- |
|  |  |  | **Estimate (95% CI)** | **P** | **Estimate (95% CI)** | **P** | **Estimate (95% CI)** | **P** | **P for test of Heterogeneity** |  |
| **BD-PRS** | ***Inter-episode remission*** | OR | 1.17 (1.09,1.26) | 8.26×10^-6^ | 1.15 (1.05,1.26) | 0.003 | **1.16 (1.10,1.23)** | **1.05×10^-7^** | 0.73 |  |
|  | ***Psychotic symptoms*** |  | 1.02 (0.95,1.11) | 0.53 | 1.21 (1.09,1.33) | 2.12×10^-4^ | 1.11 (0.94,1.30) | 0.21 | 0.01 |  |
|  | ***Comorbid anxiety*** |  | 0.87 (0.81,0.94) | 1.94×10^-4^ | 0.88 (0.79,0.99) | 0.03 | **0.88 (0.83,0.93)** | **1.60×10^-5^** | 0.88 |  |
|  | ***GAF function*** | Beta | 0.99 (0.53,1.44) | 2.24×10^-5^ | 0.59 (0.16,1.01) | 0.006 | **0.78 (0.38,1.17)** | **1.06×10^-4^** | 0.21 |  |
|  | ***GAF symptom*** |  | 0.96 (0.53,1.38) | 1.14×10^-5^ | - | - | - | - |  |  |
| **SCZ-PRS** | ***Inter-episode remission*** | OR | 0.91 (0.85,0.98) | 0.008 | 0.90 (0.82,0.99) | 0.03 | **0.91 (0.86,0.96)** | **6.98×10^-4^** | 0.83 |  |
|  | ***Psychotic symptoms*** |  | 1.19 (1.10,1.28) | 7.92×10^-6^ | 1.19 (1.08,1.32) | 7.06×10^-4^ | **1.19 (1.12,1.26)** | **2.07×10^-8^** | 0.98 |  |
|  | ***Comorbid anxiety*** |  | 1.01 (0.94,1.09) | 0.74 | 0.99 (0.89,1.11) | 0.89 | 1.01 (0.95,1.07) | 0.83 | 0.77 |  |
|  | ***GAF function*** | Beta | -0.70 (-1.15, -0.25) | 0.002 | -0.30 (-0.73,0.12) | 0.16 | -0.49 (-0.88,-0.11) | 0.01 | 0.21 |  |
|  | ***GAF symptom*** |  | -0.38 (-0.80, 0.05) | 0.08 | - | - | **-** | **-** |  |  |
| ***MDD-PRS*** | ***Inter-episode remission*** | OR | 0.84 (0.79,0.89) | 2.81×10^-8^ | 0.85 (0.78,0.93) | 2.35×10^-4^ | **0.84 (0.80, 0.89)** | **2.78×10^-11^** | 0.84 |  |
|  | ***Psychotic symptoms*** |  | 0.97 (0.90, 1.03) | 0.32 | 0.85 (0.77,0.93) | 3.92×10^-4^ | 0.91 (0.80,1.03) | 0.14 | 0.02 |  |
|  | ***Comorbid anxiety*** |  | 1.15 (1.08,1.22) | 2.41×10^-5^ | 1.15 (1.03,1.27) | 0.01 | **1.15 (1.09,1.21)** | **8.73×10^-7^** | 0.99 |  |
|  | ***GAF function*** | Beta | -0.86 (-1.26,-0.45) | 3.61×10^-5^ | -0.55 (-0.95,-0.16) | 0.006 | **-0.70 (-1.00,-0.40)** | **3.76×10^-6^** | 0.29 |  |
|  | ***GAF symptom*** |  | -0.89 (-1.27,-0.52) | 3.79×10^-6^ | - | - | - | - |  |  |

Abbreviations. PRSs: polygenic risk scores; BD: bipolar disorder; SCZ: schizophrenia; MDD: major depressive disorder; OR: odds ratio; CI: confidence interval; Logistic regression was applied in analyses for psychotic symptoms and comorbid anxiety in both cohorts. For inter-episode remission, ordinal logistic regression was applied in SWEBIC cohort and logistic regression was applied in BDRN cohort. Odds ratios (OR) and 95% confidence intervals (CI) are reported. Linear regression was applied for GAF function and GAF symptom, and Beta and 95% CI are reported. Analyses included all three PRS and were adjusted for sex, birth year, the first six population principal components, and genotyping platforms. Random-effect meta-analyses were performed combining the results from two cohorts. Estimates past significance threshold (corrected for 12 tests in meta-analyses, P<0.004) are marked in bold.

**Supplementary Table S4. Association between subphenotypes in patients with bipolar disorder and polygenic risk scores of schizophrenia, bipolar disorder, major depressive disorder, anxiety disorders, neuroticism, and educational attainment**

| **PRS** | **BD subphenotypes** | **coefficient** | **SWEBIC** | | **BDRN** | | **Meta** | | | |
| --- | --- | --- | --- | --- | --- | --- | --- | --- | --- | --- |
|  |  |  | **Estimate (95% CI)** | **P** | **Estimate (95% CI)** | **P** | **Estimate (95% CI)** | **P** | **P for test of Heterogeneity** |  |
| **BD-PRS** | ***Inter-episode remission*** | OR | 1.15 (1.07,1.23) | 9.16×10^-5^ | 1.13 (1.03,1.24) | 0.01 | **1.14 (1.08,1.21)** | **3.45×10^-6^** | 0.74 |  |
|  | ***Psychotic symptoms*** |  | 1.02 (0.94,1.10) | 0.70 | 1.20 (1.08,1.32) | 4.59×10^-4^ | 1.10 (0.94,1.29) | 0.25 | 0.01 |  |
|  | ***Comorbid anxiety*** |  | 0.88 (0.82,0.95) | 6.05×10^-4^ | 0.91 (0.81,1.02) | 0.10 | **0.89 (0.84,0.95)** | **1.63×10^-4^** | 0.66 |  |
|  | ***GAF function*** | Beta | 0.90 (0.45,1.36) | 1.05×10^-4^ | 0.57 (0.15,0.99) | 0.01 | **0.73 (0.40,1.05)** | **1.19×10^-5^** | 0.30 |  |
|  | ***GAF symptom*** |  | 0.89 (0.46,1.31) | 4.26×10^-5^ | - | - | - | - |  |  |
| **SCZ-PRS** | ***Inter-episode remission*** | OR | 0.92 (0.86,0.98) | 0.01 | 0.90 (0.82,0.99) | 0.04 | **0.91 (0.87,0.97)** | **0.001** | 0.79 |  |
|  | ***Psychotic symptoms*** |  | 1.19 (1.11,1.29) | 5.04×10^-6^ | 1.19 (1.08,1.32) | 6.51×10^-4^ | **1.19 (1.12,1.27)** | **1.23×10^-8^** | 0.98 |  |
|  | ***Comorbid anxiety*** |  | 1.00 (0.93,1.08) | 0.95 | 0.99 (0.88,1.11) | 0.84 | 1.00 (0.94,1.06) | 0.96 | 0.84 |  |
|  | ***GAF function*** | Beta | -0.63 (-1.08,-0.18) | 0.006 | -0.30 (-0.73,0.13) | 0.17 | -0.46 (-0.78,-0.13) | 0.006 | 0.30 |  |
|  | ***GAF symptom*** |  | -0.30 (-0.72, 0.12) | 0.17 | - | - | **-** | **-** |  |  |
| ***MDD-PRS*** | ***Inter-episode remission*** | OR | 0.89 (0.83,0.95) | 4.57×10^-4^ | 0.88 (0.80,0.97) | 0.01 | **0.89 (0.84,0.93)** | **9.96×10^-6^** | 0.86 |  |
|  | ***Psychotic symptoms*** |  | 1.01 (0.94, 1.08) | 0.87 | 0.85 (0.77,0.93) | 9.09×10^-4^ | 0.93 (0.78,1.10) | 0.37 | 0.01 |  |
|  | ***Comorbid anxiety*** |  | 1.08 (1.01,1.15) | 0.03 | 1.04 (0.93,1.17) | 0.46 | 1.07 (1.01,1.13) | 0.02 | 0.61 |  |
|  | ***GAF function*** | Beta | -0.40 (-0.83,0.03) | 0.07 | -0.53 (-0.95,-0.11) | 0.01 | -0.47 (-0.76,-0.17) | 0.002 | 0.67 |  |
|  | ***GAF symptom*** |  | -0.42 (-0.82,-0.03) | 0.04 | - | - | - | - |  |  |
| ***ANX-PRS*** | ***Inter-episode remission*** | OR | 0.96 (0.90,1.01) | 0.14 | 0.98 (0.90,1.07) | 0.69 | 0.97 (0.92,1.01) | 0.15 | 0.60 |  |
|  | ***Psychotic symptoms*** |  | 1.00 (0.94,1.07) | 0.98 | 0.98 (0.89,1.07) | 0.59 | 0.99 (0.94,1.04) | 0.77 | 0.65 |  |
|  | ***Comorbid anxiety*** |  | 1.03 (0.97,1.09) | 0.41 | 0.99 (0.90,1.10) | 0.91 | 1.02 (0.97,1.07) | 0.51 | 0.61 |  |
|  | ***GAF function*** | Beta | -0.26 (-0.64,0.12) | 0.17 | -0.04 (-0.43,0.34) | 0.82 | -0.15 (-0.42,0.11) | 0.26 | 0.43 |  |
|  | ***GAF symptom*** |  | -0.29 (-0.64, 0.06) | 0.11 |  |  |  |  |  |  |
| ***Neuroticism-PRS*** | ***Inter-episode remission*** | OR | 0.93 (0.88, 0.99) | 0.03 | 0.97 (0.88,1.06) | 0.46 | 0.94 (0.90, 0.99) | 0.03 | 0.54 |  |
|  | ***Psychotic symptoms*** |  | 0.94 (0.88,1.00) | 0.05 | 1.05 (0.95,1.15) | 0.33 | 0.98 (0.88,1.10) | 0.79 | 0.05 |  |
|  | ***Comorbid anxiety*** |  | 1.12 (1.05,1.19) | 2.71×10^-4^ | 1.20 (1.07,1.33) | 0.001 | **1.14 (1.08,1.21)** | **3.63×10^-6^** | 0.31 |  |
|  | ***GAF function*** | Beta | -0.83 (-1.22,-0.43) | 4.10×10^-5^ | 0.02 (-0.39,0.42) | 0.93 | -0.41 (-1.24,0.42) | 0.34 | 0.003 |  |
|  | ***GAF symptom*** |  | -0.95 (-1.32,-0.58) | 4.24×10^-7^ |  |  |  |  |  |  |
| ***EA-PRS*** | ***Inter-episode remission*** | OR | 1.17 (1.10, 1.24) | 2.34×10^-7^ | 1.17 (1.07,1.27) | 4.89×10^-4^ | **1.17 (1.11, 1.22)** | **4.50×10^-10^** | 1.00 |  |
|  | ***Psychotic symptoms*** |  | 1.11 (1.04, 1.18) | 0.002 | 1.08 (0.99,1.19) | 0.09 | **1.10 (1.04, 1.16)** | **4.84×10^-4^** | 0.70 |  |
|  | ***Comorbid anxiety*** |  | 0.91 (0.86, 0.96) | 0.001 | 0.80 (0.72,0.89) | 2.56×10^-5^ | 0.86 (0.76, 0.97) | 0.02 | 0.04 |  |
|  | ***GAF function*** | Beta | 0.86 (0.48,1.23) | 7.42×10^-6^ | 0.13 (-0.26,0.52) | 0.50 | 0.50 (-0.21,1.21) | 0.17 | 0.01 |  |
|  | ***GAF symptom*** |  | 0.68 (0.33, 1.04) | 1.30×10^-4^ |  |  |  |  |  |  |

Abbreviations. PRSs: polygenic risk scores; BD: bipolar disorder; SCZ: schizophrenia; MDD: major depressive disorder; ANX: anxiety disorders; EA: educational attainment; OR: odds ratio; CI: confidence interval; Logistic regression was applied in analyses for psychotic symptoms and comorbid anxiety in both cohorts. For inter-episode remission, ordinal logistic regression was applied in SWEBIC cohort and logistic regression was applied in BDRN cohort. Odds ratios (OR) and 95% confidence intervals (CI) are reported. Linear regression was applied for GAF function and GAF symptom, and Beta and 95% CI are reported. Analyses included all six PRS and were adjusted for sex, birth year, the first six population principal components, and genotyping platforms. Random-effect meta-analyses were performed combining the results from two cohorts. Estimates past significance threshold (corrected for 24 tests in meta-analyses, P<0.002) are marked in bold.

**Supplementary Table S5. Association between subphenotypes in patients with bipolar disorder and polygenic risk scores of schizophrenia (SCZ-PRS), bipolar disorder (BD-PRS), and major depressive disorder (MDD-PRS)** **in BD1 cases**

| **PRS** | **BD subphenotypes** | **coefficient** | **SWEBIC BD1 (N=2,287)** | | **BDRN BD1 (N=1,646)** | | **Meta** | | |
| --- | --- | --- | --- | --- | --- | --- | --- | --- | --- |
|  |  |  | **Estimate (95% CI)** | **P** | **Estimate (95% CI)** | **P** | **Estimate (95% CI)** | **P** | **P for test of Heterogeneity** |
| **BD-PRS** | ***Inter-episode remission*** | OR | 1.25 (1.12,1.39) | 4.03×10^-5^ | 1.17 (1.03,1.31) | 0.01 | **1.21 (1.12,1.31)** | **2.13×10^-6^** | 0.41 |
|  | ***Psychotic symptoms*** |  | 1.00 (0.88,1.13) | 0.97 | 1.11 (0.96,1.28) | 0.14 | 1.05 (0.94,1.16) | 0.40 | 0.26 |
|  | ***Comorbid anxiety*** |  | 0.92 (0.83, 1.03) | 0.14 | 0.91 (0.79, 1.04) | 0.17 | 0.92 (0.84,1.00) | 0.04 | 0.88 |
|  | ***GAF function*** | Beta | 1.09 (0.36,1.82) | 0.004 | 0.58 (0.05,1.11) | 0.03 | **0.77 (0.29,1.25)** | **0.002** | 0.27 |
|  | ***GAF symptom*** |  | 0.88 (0.20,1.56) | 0.01 | - | - | - | - |  |
| **SCZ-PRS** | ***Inter-episode remission*** | OR | 0.88 (0.80,0.98) | 0.02 | 0.85 (0.76, 0.96) | 0.01 | **0.87 (0.80,0.94)** | **4.21×10^-4^** | 0.68 |
|  | ***Psychotic symptoms*** |  | 1.22 (1.08,1.38) | 0.001 | 1.06 (0.92,1.22) | 0.42 | 1.14 (1.00, 1.31) | 0.05 | 0.15 |
|  | ***Comorbid anxiety*** |  | 0.96 (0.86,1.07) | 0.48 | 0.96 (0.84,1.10) | 0.54 | 0.96 (0.88,1.05) | 0.35 | 0.97 |
|  | ***GAF function*** | Beta | -1.04 (-1.76, -0.33) | 0.004 | -0.30 (-0.83, 0.24) | 0.28 | -0.63 (-1.35,0.10) | 0.09 | 0.10 |
|  | ***GAF symptom*** |  | -0.77 (-1.44, -0.10) | 0.02 | - | - | **-** | **-** |  |
| ***MDD-PRS*** | ***Inter-episode remission*** | OR | 0.80 (0.73,0.88) | 3.57×10^-6^ | 0.85 (0.76, 0.95) | 0.004 | **0.82 (0.76, 0.88)** | **5.72×10^-8^** | 0.50 |
|  | ***Psychotic symptoms*** |  | 0.99 (0.89,1.10) | 0.89 | 0.93 (0.82,1.07) | 0.32 | 0.97 (0.89,1.05) | 0.47 | 0.48 |
|  | ***Comorbid anxiety*** |  | 1.20 (1.09,1.32) | 1.99×10^-4^ | 1.12 (0.99, 1.28) | 0.07 | **1.17 (1.08,1.26)** | **5.07×10^-5^** | 0.43 |
|  | ***GAF function*** | Beta | -0.94 (-1.56, -0.31) | 0.003 | -0.64 (-1.13, -0.14) | 0.01 | **-0.75 (-1.14,-0.36)** | **1.43×10^-4^** | 0.45 |
|  | ***GAF symptom*** |  | -0.77 (-1.36, -0.19) | 0.01 | - | - | - | - |  |

Abbreviations. PRSs: polygenic risk scores; BD: bipolar disorder; SCZ: schizophrenia; MDD: major depressive disorder; OR: odds ratio; CI: confidence interval; Logistic regression was applied in analyses for psychotic symptoms and comorbid anxiety in both cohorts. For inter-episode remission, ordinal logistic regression was applied in SWEBIC cohort and logistic regression was applied in BDRN cohort. Odds ratios (OR) and 95% confidence intervals (CI) are reported. Linear regression was applied for GAF function and GAF symptom, and Beta and 95% CI are reported. Analyses included all three PRS and were adjusted for sex, birth year, the first six population principal components, and genotyping platforms. Random-effect meta-analyses were performed combining the results from two cohorts. Estimates past significance threshold (corrected for 12 tests in meta-analyses, P<0.004) are marked in bold.

**Supplementary Table S6. Association between subphenotypes in patients with bipolar disorder and polygenic risk scores of schizophrenia (SCZ-PRS), bipolar disorder (BD-PRS), and major depressive disorder (MDD-PRS)** **in BD2 cases**

| **PRS** | **BD subphenotypes** | **coefficient** | **SWEBIC BD2 (N=1,704)** | | **BDRN BD2 (N=795)** | | **Meta** | | |
| --- | --- | --- | --- | --- | --- | --- | --- | --- | --- |
|  |  |  | **Estimate (95% CI)** | **P** | **Estimate (95% CI)** | **P** | **Estimate (95% CI)** | **P** | **P for test of Heterogeneity** |
| **BD-PRS** | ***Inter-episode remission*** | OR | 1.08 (0.96,1.22) | 0.19 | 1.13 (0.96,1.34) | 0.14 | 1.10 (1.00,1.21) | 0.05 | 0.66 |
|  | ***Comorbid anxiety*** |  | 0.94 (0.84,1.07) | 0.36 | 0.88 (0.70,1.11) | 0.27 | 0.93 (0.83,1.04) | 0.19 | 0.58 |
|  | ***GAF function*** | Beta | 0.53 (-0.18,1.25) | 0.14 | 0.38 (-0.34,1.10) | 0.30 | 0.46 (-0.05, 0.97) | 0.08 | 0.77 |
|  | ***GAF symptom*** |  | 0.76 (0.09,1.42) | 0.03 |  |  | - | - |  |
| **SCZ-PRS** | ***Inter-episode remission*** | OR | 0.94 (0.84,1.05) | 0.24 | 0.97 (0.83,1.15) | 0.75 | 0.95 (0.86,1.04) | 0.25 | 0.70 |
|  | ***Comorbid anxiety*** |  | 1.03 (0.91,1.16) | 0.61 | 1.15 (0.91,1.44) | 0.23 | 1.06 (0.95,1.17) | 0.32 | 0.41 |
|  | ***GAF function*** | Beta | -0.45 (-1.16, 0.26) | 0.22 | -0.24 (-0.96, 0.47) | 0.51 | -0.35 (-0.85, 0.16) | 0.18 | 0.69 |
|  | ***GAF symptom*** |  | -0.09 (-0.75, 0.57) | 0.79 | - | - | **-** | **-** |  |
| ***MDD-PRS*** | ***Inter-episode remission*** | OR | 0.88 (0.79,0.98) | 0.02 | 0.84 (0.72,0.97) | 0.02 | **0.87 (0.79, 0.94)** | **0.001** | 0.56 |
|  | ***Comorbid anxiety*** |  | 1.06 (0.95,1.18) | 0.33 | 1.13 (0.91,1.40) | 0.28 | 1.07 (0.97,1.18) | 0.17 | 0.60 |
|  | ***GAF function*** | Beta | -0.36 (-1.00,0.29) | 0.28 | -0.11 (-0.76,0.53) | 0.73 | -0.23 (-0.69, 0.22) | 0.31 | 0.60 |
|  | ***GAF symptom*** |  | -0.75 (-1.35, -0.15) | 0.02 | - | - | - | - |  |

Abbreviations. PRSs: polygenic risk scores; BD: bipolar disorder; SCZ: schizophrenia; MDD: major depressive disorder; OR: odds ratio; CI: confidence interval. Logistic regression was applied in analyses for comorbid anxiety in both cohorts. Psychotic symptoms during mood episodes was not tested because BD2 cases did not have this subphenotype. For inter-episode remission, ordinal logistic regression was applied in SWEBIC cohort and logistic regression was applied in BDRN cohort. Odds ratios (OR) and 95% confidence intervals (CI) are reported. Linear regression was applied for GAF function and GAF symptom, and Beta and 95% CI are reported. Analyses included all three PRS and were adjusted for sex, birth year, the first six population principal components, and genotyping platforms. Random-effect meta-analyses were performed combining the results from two cohorts. Estimates past significance threshold (corrected for 9 tests in meta-analyses, P<0.005) are marked in bold.

**Supplementary Table S7. Association between subphenotypes in index person with bipolar disorder and lifetime diagnosis of three major psychiatric disorders in the first- and second-degree relatives**

| **Psychiatric disorders in relatives** | **Subphenotypes in index BD person** | **OR/Beta (95% CI)** | **P** |
| --- | --- | --- | --- |
| **SCZ vs BD** | |  |  |
|  | **GAF-Function** | **-1.79 (-2.60, -0.98)** | **9.35×10^-6^** |
|  | **GAF-Symptom** | **-1.35 (-2.10, -0.60)** | **5.00×10^-4^** |
|  | **Comorbid Anxiety** | **1.11 (1.04, 1.18)** | **0.001** |
|  | **Psychotic symptom** | **1.23 (1.14, 1.32)** | **1.33×10^-8^** |
| **MDD vs BD** | |  |  |
|  | **GAF-Function** | **-1.05 (-1.55, -0.55)** | **2.59×10^-4^** |
|  | **GAF-Symptom** | **-1.07 (-1.54, -0.61)** | **4.46×10^-5^** |
|  | **Comorbid Anxiety** | **1.32 (1.27, 1.38)** | **<1×10^-10^** |
|  | Psychotic symptom | 0.95 (0.90, 1.00) | 0.04 |

Abbreviations. GAF-Function: global assessment of functioning including function dimensions; GAF-Symptom: global assessment of functioning including symptom. BD: bipolar disorder; SCZ: schizophrenia; MDD: major depressive disorder; OR: odds ratio; CI: confidence interval; Logistic regression was applied in analyses for psychotic symptoms and comorbid anxiety. Linear regression was applied for GAF function and GAF symptom and Beta as the regression coefficients. Models were adjusted for sex, categorical year of birth and degree of biological relatedness. For anxiety and psychotic symptom (which was extracted from the Swedish National Registries), models were additionally adjusted for anxiety or psychotic symptoms in the relatives. Results exceeding multiple testing corrected significance threshold (8 tests, P < 0.006) are marked in bold.
